# Abuse of power in the disciplinary actions of a state psychology licensing board: inequitable outcomes and early career psychologists

**DOI:** 10.3389/fpsyg.2023.1184528

**Published:** 2023-09-14

**Authors:** Sonya C. Faber, Edward Wu, Amy Bartlett

**Affiliations:** ^1^School of Psychology, University of Ottawa, Ottawa, ON, Canada; ^2^Faculty of Civil Law, University of Ottawa, Ottawa, ON, Canada; ^3^Department of Religious Studies, University of Ottawa, Ottawa, ON, Canada

**Keywords:** early career psychologists, state boards, disciplinary action, equity, governance, psychology licensing boards

## Abstract

The field of psychology has established high professional standards which have become a cornerstone of the practice of psychology. However, powerful boards tasked with administering these standards can operate with little oversight, making it difficult to monitor whether these institutions are operating in a fair and impartial way. In particular, early-career psychologists who have less experience and power in their initial years of independent practice may be singularly vulnerable as they have relatively little experience to navigate the profession, including fielding complaints that may be made against them to a licensing board. While it is essential to ensure early-career psychologists are upholding their commitments to the practice, there are risks in policing their activities without orienting toward growth, learning, and professional development. Even the smallest disciplinary action may never be expunged from a psychologist’s record, resulting in long-term implications for insurance coverage, reputation and future professional viability in the field. Overly-punitive approaches can be distressing or even traumatizing. In this paper, we examine disciplinary actions of the Kentucky Board of Examiners of Psychology (KBEP) from the years 2000 to 2020 (*N* = 65) to determine the methodology by which the Board administers its oversight function. We analyze the nature of the discipline received (fines, suspensions, continuing education, supervision) revealing a two-tiered system of punishments, and provide context regarding the nature of the disciplinary process and its impacts. We report on qualitative interviews of early career psychologists subject to disciplinary actions by the Board, and psychologists who supervised early career psychologists investigated by the Board. We compare legislation governing KBEP and make comparisons to the workings of licensing boards in three other states. Using these findings, we make recommendations for revisions to the applicable legislation and administrative processes of the Board to establish an improved balance between public safety, the well-being of new psychologists, equity considerations such as race, and the development of the practice of psychology in Kentucky. This work brings to light previously unexamined injustices that can knowingly or unknowingly be perpetuated by licensing Boards, and can be used to inform the creation of more just, balanced and inclusive professional Boards.

## 1. Introduction

The field of psychology has established high professional standards for the people who choose to enter the practice, and these standards help to regulate and hold accountable those who have dedicated their careers to caring for people navigating relational and mental health needs. Given the intimate and essential character of this work, accountability and ethical conduct has become one of the cornerstones of the practice of psychology. However, the professional oversight, accountability and rigor demanded from psychology professionals should also inform the conduct of those providing that professional oversight. In short, who is watching the watchers?

To explore this question, in this paper we examine the methodology by which a single state Board– the Kentucky Board of Examiners of Psychology (KBEP or “the Board”) – administers disciplinary actions. We uncover how early-career psychologists have been particularly harmed by the very body meant to oversee their professional development. We outline some of the challenges faced by early-career psychology professionals in the state of Kentucky. We describe disciplinary actions of KBEP including their outcomes and implications, while offering an analysis of the methodology of the Board based on their rulings in previous cases. We also note the lack of diversity of the Board and how this may contribute to an unacknowledged culture of discrimination and inequitable treatment.

We conclude by making recommendations to help ensure the Board is more accountable for their work and has mechanisms in place to ensure equitable treatment of all psychologists operating in the state. Understanding the power dynamics between accountability boards and the people they are meant to hold accountable is an under researched topic in academia generally, and in the psychology profession in particular. It is hoped that this case study can also inform the development of more equitable psychology licensing Board practices across the country. The methods introduced in this paper can be used to make similar assessments in other States where power is wielded without the opportunity to monitor, evaluate and adjust practices to best serve the public as well as the evolving field and practice of psychology.

### 1.1. Purpose of the Kentucky board of examiners of psychology

The purpose of KBEP^1^ revolves around two main functions: (1) managing the administration and oversight for licensing professional psychologists in Kentucky; and (2) monitoring client needs and public safety regarding the practice of psychology in the state. To fulfill these functions, the Board engages in regular meetings to review new and ongoing issues, verifies the qualifications of incoming psychologists, conducts formal hearings, and implements disciplinary actions when necessary. It also makes recommendations to update the laws, policies and procedures governing the practice in Kentucky to ensure fairness and equality.

In 1948, Kentucky passed the “Certification of Clinical Psychologists” law and became the third or fourth state to recognize psychology as a regulated profession (Schuster, 2011). The Kentucky Board of Examiners of Psychology (KBEP), initially consisting of 5 doctoral-level psychologists, was created to help regulate the practice on behalf of the state. In 1964, the statute was revised to limit who could be called a “Psychologist” to only individuals who had a doctoral degree or equivalent in psychology (Schuster, 2011). In the mid-1970s, the Board implemented more restrictive policies, refraining from issuing certificates for Autonomous Functioning which made it difficult for Master’s-level psychologists to practice independently (Schuster, 2011).

Since that time, the composition of the Board has been updated (HB 330/CI, 1992): they now have 8 psychologists and 1 citizen at large for a total of 9 members (Kentucky Board of Examiners of Psychology, 2022). However, in the state of Kentucky, none of the psychologist seats have ever been held by a person of color, and currently, neither the psychologists nor the citizen at large is a person of color (Kentucky Board of Examiners of Psychology, 2022). There are also no board seats occupied by early-career psychology professionals.

### 1.2. Early career psychologists

The American Board of Professional Psychology defines an early-career psychologist (ECP) as someone who already has their doctoral degree but earned it less than 10 years ago (American Board of Professional Psychology [ABPP], 2022). Alternatively, the Kentucky Psychological Association (n.d.) defines early-career psychologists as “someone who has completed a master’s or doctoral degree within the last 7 years.” For the purposes of this paper, we apply the KPA 7-year window after degree completion to qualify as an ECP.

Like all new practitioners to their chosen field, for early-career psychologists there is a learning curve as people get practical experience applying the theory and research they have gained during their studies and translate the knowledge into successful day-to-day practice. These psychologists are in need of monitoring and mentorship as they navigate their entry into the profession, as well as receiving understanding and navigating the inevitable failures that come as they learn and grow into mature clinical practitioners.

Early-career psychologists represent the future of the field and provide unique perspectives to the discipline and practice of psychology (Hall and Boucher, 2008). Without new psychologists joining the practice, the future of psychological practice is vulnerable not only to decreasing membership but ultimately decreasing relevance (Forrest, 2012). Early-career psychologists evolve the current state of psychology in order to ensure the practice can meet the demands of the changing needs of its members and the public (Forrest, 2012; Smith et al., 2012). The psychological issues confronting upcoming generations include the internet, globalization, COVID-19, and rising diversity, many of which are not the same issues that led to the need for a psychologist in past generations. In fact, early-career practitioners lead the way in our understanding of how to help clients navigate these concerns due to their contemporary and up-to-date training as well as shared lived experience with younger clients.

### 1.3. Ramifications of accusations of misconduct and disciplinary actions

After many long years of study and practice to enter the field, receiving formal notice of a licensing complaint can be extremely distressing for practitioners at any time in their career (Kirkcaldy et al., 2020). Shock and surprise are common reactions, and fear and anxiety are pervasive among those accused of malpractice or errors (Schoenfeld et al., 2001). A primary reason for severe anxiety revolves around potentially losing the ability to earn an income. For some, this type of disciplinary notice early in their practice can be traumatic. In a study of psychologists’ experiences of a misconduct complaint, one accused practitioner shared: “I think I was traumatized. it consumed me. I never want to go through it again, in my life. it is beyond devastation” (Kirkcaldy et al., 2020, p. 405). Complaints of professional misconduct, whether substantiated or not, can cause a loss of professional confidence and capacity. Speaking about their experience of receiving a complaint, one person said: “I felt worthless, [like] I cannot do this job, I am totally incompetent. I need to relook at the degree whether this is supposed to be my life, or should I be doing something else. I questioned everything that I did” (Kirkcaldy et al., 2020, p. 407).

Early-career psychologists may be particularly susceptible to this sort of traumatization and loss of confidence when faced with accusations of professional misconduct. However, the risk of traumatization can increase when these harsh accusations are received from the regulatory body that is meant to oversee their practice, and especially without some basic supportive guidance about the administrative process to understand what is happening, why it is happening, and that they are going to be accompanied collaboratively and compassionately to address the situation. This effect is amplified by the negative professional exposure this type of early-career reprimand can bring and the increasing availability of disciplinary information online (e.g., Szalados, 2021). Table 1 below outlines the possible disciplinary actions that the KBEP can take, and even a public reprimand can have long-term negative implications for a psychologist’s ability to get insurance coverage, receive licensing approval in other jurisdictions, gain professional society membership, secure clients, and affect their overall reputation within the field (Jesilow and Ohlander, 2010; Coy et al., 2016; Szalados, 2021). Despite the enormous impact of even the mildest of these disciplinary actions, the reality is that most of the accusations of potential misconduct by early-career psychologists (as with any early-career professional) are in reference to actions committed through honest error or misunderstanding, not malicious intent or gross negligence (Novotney, 2020). In contrast to policing serious ethical code violations that are a result of willful misconduct, a different approach to support people new to the profession and teach and nurture them as they grow into their role as a well-informed and ethical member of the psychological community would improve the nature of this process.

**TABLE 1:** Disciplinary actions utilized by the Kentucky board of psychological examiners.

Disciplinary actions the KBEP can invoke	What it entails
Probation	This is a disciplinary action that entails supervised practice, where the license can be revoked if the board finds that the imposed conditions are not being followed.
Reprimand	This is a disciplinary action by the Board, and it does appear on the psychologist’s public record. It seems to signify that the Board has formally and publicly recognized the psychologist’s wrongdoing and the settlement agreement equates to the psychologist “agreeing to the wrongdoing.”
Private admonishment	This is not a disciplinary action and is used for statistical purposes. If a private admonishment is invoked, it is not publicly accessible but remains in the psychologist’s file and can be used as evidence if new issues arise in KY.
Suspension of license	This is a disciplinary action by the Board, and during the suspension the psychologist cannot practice.
Revocation or refusal to renew license	The license is lost and the psychologist can no longer practice as long as the license remains revoked. Three years after the date of revocation, a person can appeal to the Board for reinstatement (319.082), unless indicated otherwise in the order.
Fine	If a fine is invoked, it cannot exceed $1,000 per violation. The fine stays on their disciplinary record. The purpose of a fine is not typically punitive, rather to reimburse the board for their investigation and administrative costs. Fines however also have been levied to redress court costs or compensate plaintiffs and in such cases could be considered punitive.

The emotional turmoil that can result from accusations of professional misconduct intersect both the personal and professional life of a psychologist as it casts doubt on their abilities, credibility, competency, and integrity as an individual and a practitioner (Thomas, 2005; Kirkcaldy et al., 2020). The stress of trying to navigate the accusations being made against them, while also preserving their reputation and high-level of client services while they continue to practice, can have a severe negative impact on their emotional and psychological state (Thomas, 2005). Accusations of professional misconduct can also result in significant issues such as low self-esteem, anxiety, and depression, all of which can lead to impaired professional abilities, judgment, and acute distress (Thomas, 2005).

### 1.4. Psychology in Kentucky

Psychologists are fundamental to the vital backbone of mental health services nationwide. In addition to their crucial role in mental health care delivery, they provide leadership in community mental health centers and hospitals. Psychologists in primary health care may administer and interpret clinical assessments. Important psychological tests and assessments include determining functioning and aptitude, making diagnoses, and identifying treatment needs for complex psychopathologies. Outside of medical settings, psychologists spend considerable time teaching and training all types of mental health care providers. They serve as mentors in post-secondary institutions – contributing to success within academia and institutional culture. Additionally, through research, these professional psychologists find evidence-based solutions to mental health disorders, and sometimes they venture beyond clinical settings and contribute to policy and legal work. For the most part, psychologists receive high quality education and are well-prepared for their varied professional roles (e.g., Mikail and Nicholson, 2019).

Although the APA estimates that 106,000 psychologists are licensed in the United States, the distribution and subsequent access to these psychologists by the public is quite geographically heterogenous (Lin et al., 2020). In some areas with high accessibility there are up to 3,600 in a county, and in other less well-prosperous locales, there are zero psychologists and no access to mental health resources. Kentucky has the fourth highest number of counties (120) among US states and counts the most number of counties with zero psychologists (Lin et al., 2016). Across the US, the areas with the highest access to licensed psychologists include the Miami and Seattle areas, California, and the Northeast. There is a notable dearth of access in the heart of the nation, and in Kentucky specifically.

According to the APA, in 2018, there were approximately 785 active doctoral-level licensed psychologists in Kentucky, which are a subset of the approximately 1,120 clinical, counseling and school psychologists in the state (American Psychological Association, 2020; U. S. Bureau of Labor Statistics, 2022). The concentration of licensed psychologists was low compared to the nation as a whole, with only 17.5 per 100,000 persons, compared to 32.0 per 100,000 in the US as a whole, notably lower than the national average. This number is not adequate to meet the demand for mental health services in Kentucky, as 22.14% of adults experienced mental illness (of any kind) in 2020 in Kentucky, which was higher than the national average (19%) (American Psychological Association, 2020). In addition, approximately 4.65% of the Kentucky population ages 12 and older had alcohol use disorder in the previous year which although lower than the national average (5.37%), did not eclipse the alarmingly high number of drug overdoses. The drug overdose mortality rate in Kentucky in 2018 came to 30.9 deaths per 100,000, compared to only 19.8 deaths per 100,000 nationally. Data shows that many counties, particularly in the south and east of the state, have a lack of professional psychologists despite a high need for them (American Psychological Association, 2020).

### 1.5. Purpose of this investigation

Some contributors to this report have practiced in Kentucky and feel a great affection for the state and its people. They have served as members of the Kentucky psychological community and share the concerns expressed by others that disciplinary measures taken by the Board against psychology credential holders have done damage to the practice of psychology in Kentucky, and have been at times overly harsh, inconsistent, aggressive, or lacking in transparency. As an example, consider the Board’s legal response to the 35-years running advice column of the syndicated board-certified psychologist, John Rosemund (NC), who was threatened and censored by KBEP because they disagreed with his perspectives. Institute for Justice Attorney Paul Sherman opined that, “Kentucky’s definition of the practice of psychology is so broad, and the board has demonstrated itself to be so aggressive, that we don’t know what they think the limits on their power are” (Burrows, 2013). Dr. Rosemond sued the Kentucky Psychology Board, charging them with attempting to suppress his First Amendment rights. They refused to back down, and in October 2015 ended up losing the case Rosemond had brought against them in the courts. Consider this was a prominent esteemed psychologist with enough resources and reputation to sue and resist the Board’s overreach in order to enforce his rights. However, most psychologists, and especially early-career psychologists, do not have the power or resources to engage in such a battle.

It is hoped that an analysis of the disciplinary methodology of the Board can shed light on this issue and ensure that improvements are made to the functioning of the Board. Given the relatively low numbers of psychologists in the state, it is of concern that new psychologists may be deterred from careers in Kentucky if the risks are deemed high and the psychological community unwelcoming. The purpose of this paper is to better understand the disciplinary procedures utilized by the Board, statistically assess if these measures have been applied consistently, and elucidate their effect on early-career psychologists. Finally, we make recommendations for improvement to eliminate potentially arbitrary implementation of punishment and better balance the needs of new psychologists with the public safety for the greater public good, with an eye to considerations of fairness, accountability and equity.

## 2. Materials and methods

This investigation utilized both quantitative (licensing board records) and qualitative (interviews) as data, as described in the following sections. The methods utilized were approved by the University of Ottawa’s Research Ethics Board (REB).

### 2.1. Quantitative

The primary source of data for this investigation was the disciplinary records posted on KBEP website from 2000 to 2018 (*N* = 63), in addition to records obtained through an open records request from 2019 to 2020 (*N* = 2) for a total of 65 records. We read each disciplinary report and extracted the data of interest. Variables examined included: Severity of KBEP Accusations; Early career status of psychologist (binary); Months of license suspension; Months of probation; Fine/Payment amounts; License remanded (binary); License suspended/revoked (binary); Ethnic/Racial minority status (binary); and gender.

Some of the items were difficult to code due to inconsistencies in Board orders and missing documentation. For the category of “Months of license suspension” it was unclear how to rate revocations, as the Kentucky code allows people with revoked licenses to reapply after 3 years. In these cases, we rated “Months of license suspension” at 36 months (unless the order specified otherwise), and added an additional 4 months for the application process which can be expected to be more complicated given the psychologist’s disciplinary history (total 40 months). In some cases, licenses were revoked permanently, in which case Months was entered as 150 (∼13 years) – 30 months more than the longest revocation that included a stated option to later reapply (10 years).

Severity of harm/risk to clients was rated on a scale of 0–5 by the first and third authors (senior doctoral-level health scientists), based on the details of the case, with issues related to failure in administrative matters receiving a low score (0 or 1), issues surrounding incompetent practice scoring in the middle (about a 3), and issues involving abuse or high risk of harm to clients receiving the highest score (5).

Demographics (White/Person of Color, gender) were determined based on the psychologists’ names, gender pronouns, and publicly available information on the internet. No rating was made for racial status when race could not be determined.

The statistical analysis conducted included simple descriptives (means and standard deviations), correlations between disciplinary outcomes and severity of KBEP accusations (Pearson and point biserial), and *T*-tests and Chi-Square tests to compare variables of interest between ECP psychologists and mid/late career psychologists. Data were analyzed using SPSS version 24.

### 2.2. Qualitative

To better understand the qualitative aspects of being investigated by KBEP, we conducted interviews with three (3) early-career psychologists who had been investigated by the Board, and two (2) supervisors of early-career psychologists who themselves had also been investigated by the Board in conjunction with their supervisees. Two of those interviewed later withdrew their approval due to fear of retaliation by KBEP. The authors used public records and word of mouth to recruit the psychologists. The interviews were recorded and then transcribed verbatim using the transcription feature provided by Zoom or Microsoft Teams, and then verified/corrected by a research assistant. Summaries of the experiences were produced from the interviews and presented as case studies. These case studies were supplemented and confirmed with related documentation from KBEP. Based on our observations and conversations with Kentucky psychologists, including many in governance positions, and the literature (e.g., Kirkcaldy et al., 2022), these accounts are to be considered examples of typical experiences interacting with KBEP and their impact on the accused.

## 3. Results/findings

### 3.1. Disciplinary case demographic data

By 2018, 785 licensed practicing psychologists could be found in Kentucky. If the national prevalence of Black psychologists follows the national average (4.2%) there should be about 34 Black psychologists practicing in Kentucky (American Psychological Association, 2020; U. S. Bureau of Labor Statistics, 2022). As noted, it was difficult to obtain information about the ethnoracial identity of our study subjects as this information was not included in the data available from the state. As such we were only able to provide information on 71.2% of files, and the vast majority were White. This likely speaks to the demographics of psychologists in Kentucky over the 23-year study period. We also noted that many cases emerged out of complaints related to work in legal cases, which we also enumerate below. See Table 2 for details.

**TABLE 2:** Subject descriptives.

	*N*	Total	Percent
Early career psychologists	22	64	34.4%
Women	22	65	33.8%
People of color	2	45	4.4%
Involved legal issues/family court	17	61	29.5%

In our review of the disciplinary data, we noted six primary types of allegations actioned upon by the Board, which are loosely in keeping with categories found by Association of State and Provincial Psychology Boards (2022). These are not mutually exclusive as most had allegations in more than one area. The main areas that incurred disciplinary actions were as follows:

1. Failure to maintain Adequate Records/Administrative issues (24.2%)
2. Unprofessional Conduct (87.1%)
3. Improper or inadequate supervision of trainees (9.7%)
4. Misrepresentation of credentials (22.6%)
5. Incompetence/Practicing psychology poorly (51.6%)
6. Exploitation/Physical or sexual abuse of clients (24.2%)

There were a number of different types of disciplinary actions taken toward psychologists, which included some or all of the options listed in Table 2. See Table 3 for details.

**TABLE 3:** Various disciplinary actions.

Disciplinary actions	*N*	Total	Percent	Mean	SD
License suspension (not incl. 10 revoked)	17	54	31.5%	7.74 (months)	19.00
License suspended or revoked	29	64	45.31%	NA	NA
License probation	30	54	54.55%	11.49 (months)	16.57
Requires supervision	30	64	46.88%	36.44 (hours)	61.33
Requires continuing education	19	64	29.69%	2.80 (hours)	5.45
Must pay fines/costs ($)	44	60	73.33%	$1572.36	2322.80
License reprimanded	19	64	30.16%	NA	NA

Next, we compared ECP to Mid/Late Career Psychologists on several key variables, including demographics and severity of disciplinary actions. For the most part, ECP’s received more punishments but had less severe offenses. ECP were given significantly more hours of continuing education than their Mid/Late career counterparts (*p* = 0.048), but other comparisons did not reach significance. See Table 4 for details.

**TABLE 4A:** Comparative findings.

	Psychologist stage	*N*	Mean	Std. dev.
Months’ probation	ECP	18	13.33	18.81
	Mid/late career	37	10.59	15.56
Months suspension (including revocation)	ECP	22	24.05	40.44
	Mid/late career	42	20.40	43.97
Fine/cost amount ($)	ECP	21	1099.29	1528.70
	Mid/late career	39	1827.10	2637.58
Hours of education*	ECP	22	4.66	7.10
	Mid/late career	42	1.83	4.12
Total supervision months (hrs/week × no. mos.)	ECP	22	11.18	17.40
	Mid/late career	42	7.00	12.01
Total severity of offense score (rating 1–8)	ECP	22	3.41	2.68
	Mid/late career	38	3.68	2.19
Harm risk to clients (rating 0–5)	ECP	21	2.14	2.15
	Mid/late career	39	2.41	1.50

*p < 0.05.

**TABLE 4B:** Comparative findings – crosstabs.

		No		Yes	
		***N***	**%**	***N***	**%**
License suspended/revoked	ECP	11	50.0%	11	50.0%
	Mid/late career	24	57.1%	18	42.9%
Settlement agreement	ECP	1	4.8%	20	95.2%
	Mid/late career	6	16.2%	31	83.8%
Reprimanded	ECP	16	72.7%	6	27.3%
	Mid/late career	29	69.0%	13	31.0%
Legal/family court involvement	ECP	16	72.7%	6	27.3%
	Mid/late career	26	68.4%	12	31.6%

### 3.2. Harm/risk and severity of offense by disciplinary action

Our ratings of harm/risk to clients were positively correlated with independent ratings of overall severity of offense (*r* = 0.74, *p* < 0.001). Further, harm/risk to clients was moderately but significantly correlated to months of probation, months of suspension, and whether the license was revoked or suspended. However, there was no correlation between harm/risk to clients or severity of offense and fine amounts, hours of CE required, total hours of supervision required, or if final judgments listed the license as being remanded. See Table 5 for details.

**TABLE 5:** Pearson and point biserial bivariate correlations.

		Harm/Risk to clients	Overall severity of offenses
Months of probation	Correlation	0.370**	0.261
	Sig. (2-tailed)	0.006	0.061
Months suspension (including revocation)	Correlation	0.379**	0.189
	Sig. (2-tailed)	0.003	0.148
License suspended/revoked (binary)	Correlation	0.358**	0.422***
	Sig. (2-tailed)	0.005	(0.001
Fine/cost amount ($)	Correlation	0.200	0.143
	Sig. (2-tailed)	0.132	0.284
Hours of education	Correlation	−0.106	0.049
	Sig. (2-tailed)	0.414	0.709
Total supervision hrs (weeks × no. mos.)	Correlation	0.194	0.207
	Sig. (2-tailed)	0.135	0.110
Reprimanded (binary)	Correlation	0.048	−0.085
	Sig. (2-tailed)	0.713	0.517

*N* = 53–61 due to missing data.

**p < 0.01 and ***p 0.001 (two tailed).

### 3.3. Interview data

Included here are summarized accounts from psychologists interviewed about their experiences with KBEP. To help preserve anonymity, the names of the participants and some non-relevant details are changed. Below is a summary of their experiences and how they were impacted.

#### 3.3.1. Early career psychologist accused of practicing without a license

*Case Study A:* Andrea started as an assistant professor in a large urban university, where her work was focused on the mental health of marginalized people. She was in the process of applying for a temporary license in Kentucky. According to the regulations, there is a grace period of time in which Andrea could practice psychology without having a temporary license.

Following the regulations for Kentucky, Andrea made a plan for supervision with a licensed psychologist for her clinical hours. Eventually, the supervisor told Andrea that they were missing some paperwork for Andrea’s temporary license, and she needed to apply for it. Andrea sent in her application with the appropriate paperwork. For 5 months, she did not receive any correspondence from the Board. During this period, Andrea emailed and phoned the Board continuously in hopes of receiving a response. Eventually, Andrea learned that her application was incomplete, that she was practicing without a license, and that the Board would commence an investigation. Andrea promptly turned in additional documents that she had been unaware of, but nonetheless an investigation ensued. To Andrea’s relief, the investigator had found that she was not at fault because she had contacted the Board so many times and within a reasonable timeframe where the Board could have replied to her. Nonetheless, she found the process distressing and punishing. Although she was not at fault, the Board decided to punish her by giving her a reprimand in the form of a private admonishment and not to count the year of supervised hours that Andrea had completed, forcing her to repeat the supervised year. In conclusion, Andrea stated that this experience was “probably one of the major reasons why I left the state of Kentucky as because I had such a terrible experience at the Board.”

The situation set Andrea back academically, professionally, and financially. Andrea was not paid for her services because she was being supervised. Because of experiences like this, a valuable asset for the university and an important voice for the trans community in the state was lost. Andrea moved away as soon as she could, asserting, “I’m never going back to Kentucky.” She later found a new position at a top university in another state.

#### 3.3.2. Disciplined for favorable assessment of black father

*Case Study B*: The following experience of a supervisor (Francesca) and her postdoctoral supervisee (Claire) was first recounted by Claire’s former supervisors, and then verified with legal records and additional sources. Claire was an associate director of a mental health clinic. During her time as a postdoc, under supervision, Claire completed a parental fitness report for a low-income Black father who was seeking visitation of his daughter from his ex-wife – a White woman who had since become affluent. The ex-wife was able to use her financial resources to create legal challenges to all of the father’s attempts to have a relationship with their daughter, which was facilitated by advancing unmerited stereotypes representing known biases against Black fathers in family court (e.g., Donohue, 2020). Based on the records of the case and a thorough assessment of the father, Claire and her supervisor wrote a culturally-informed parental fitness report that was favorable to granting parental visitation. As a result, the ex-wife hired a senior forensic psychologist expert to suppress the report findings through legal challenges. After making several threats against Claire and demanding she rescind her report, the ex-wife and her hired psychologist filed a Board complaint against Claire. They claimed Claire’s report was wrong and reckless and that Claire, being an early career psychologist, was unqualified to have made a determination of parental fitness. The ex-wife claimed the report made her feel suicidal.

The Board conducted an inadequate investigation whereby the investigator interviewed the White mother but not the Black father, then included the mother’s salacious and unsubstantiated accusations against the father in the report to the Board. These accusations were anti-Black stereotypes guaranteed to resonate with White individuals socialized into Southern culture who held unchecked biases. The Board accepted this incomplete account as fact and disciplined Claire, who was fully licensed by this time. Disciplinary actions included rescinding Claire’s ability to supervise students and placing her under supervision for a year. Initially, Claire was told by the Board that she could choose her own supervisor but then the Board changed their mind and appointed a sitting member of KBEP. Therefore, in a conflict of interest, Claire was supervised by a Board psychologist who had been involved in her case and would ultimately determine if Claire had successfully completed the supervision process, putting pressure on Claire to concur with the Board’s version of events. Claire was also ordered to pay weekly supervision fees to the Board-appointed/Board member psychologist, putting her both in further financial distress and creating unethical financial incentives for the supervisor. Claire was traumatized by the experience and left in debt from legal expenses as she defended herself from these accusations.

Shortly after Claire was disciplined, KBEP began an attack against her supervisor, Francesca. Francesca had provided the initial oversight of Claire’s work due to her expertise in multicultural psychology. She was no longer licensed in KY, having moved to another state years ago, but in an unprecedented maneuver, the Board began actions to retroactively revoke Francesca’s license. The Board had never pursued such an extreme penalty against anyone for alleged inadequate supervision, and never toward someone who was not license holder in the state. The Board also broke several of their own rules in an attempt to punish her (e.g., using Claire’s inadequate investigator’s report as a basis for their actions rather than doing their own investigation of Francesca).

As a Black person, Francesca felt she was being targeted by the all-White Board due to her race. She also believed that Claire had been disciplined in part because she refused to implicate Francesca and because Claire was part of a stigmatized religious faith. Francesca was not licensed in Kentucky nor practicing there, and thus posed no risk to the people of Kentucky. Based on state regulations, the Board could have opted to bar Francesca from licensure in Kentucky should she ever reapply in that state, and as such their crusade from afar felt pointedly vindictive. If the Board succeeded in their machinations to retroactively revoke Francesca’s license, Francesa would no longer be able to do her much-needed work for the courts in assessing people of color who were suffering from trauma due to discrimination. She also stood to potentially lose her ABPP certification, Interjurisdictional Practice Certificate, professional memberships, and face an investigation in the states where she was licensed in good standing.

#### 3.3.3. Early career psychologist punished for no reason at all

*Case Study C*: Daria was an early-career child psychologist who had been practicing under supervision at a local outpatient clinic when she received a letter from the Board accusing her of practicing without a license 2 years prior. The letter, received the week before Thanksgiving, was frightening and confusing as Daria had submitted all the proper paperwork within the necessary time frame required by the Board. The Board also sent a separate letter to her subsequent supervisor, Francesca, accusing the supervisor of encouraging Daria to practice without a license and threatening disciplinary action. Daria was bewildered, “You [the Board] are investigating myself and my supervisor, who wasn’t even my supervisor at the time.” The Board could have easily cleared this matter by reviewing their own files or with a phone call to Daria. She was completely innocent of any wrongdoing. After some months of no response or updates about her case, Daria reached out to the Board administrator and was told that “somehow, your case had not been voted on yet at the board meeting.” She said, “They had forgotten about my complaint.” Nonetheless, in a miscarriage of justice, she was given a private admonishment by the Board that was made part of her permanent file.

It was odd to Daria that her original supervisor of record (a White man) was not also sent a threatening Board letter, as he had provided oversight for her during the majority of her unlicensed practice while under the allowable grace period. She suspected that the attack was actually an attempt by the Board to find fault with her new supervisor due to racial bias. Daria subsequently moved away from Kentucky. Her feelings of mistrust and uncertainty with the Board stops her from coming back to Kentucky.

### 3.4. Quantitative findings – a two-tiered system

The assessment of 65 disciplinary cases heard by KBEP between the years 2000 and 2020 allowed us to examine the methodology by which the Board is disciplining members, to locate any inconsistencies, and to impute a possible reason for these inconsistencies. It is notable that a high percentage of cases involve children or child custody, as these types of cases have long been noted to be particularly inclined to result in disciplinary complaints (Kirkland and Kirkland, 2001).

We found a positive correlation between harm/risk to clients and independent ratings of overall severity of offense, which is what one would expect to find if the Board was operating in a just and impartial way in its disciplinary responsibilities. Additionally, the significant correlation between risk/harm to clients and punishments having to do with *duration of exclusion from active practice* (months of probation, months of suspension and revocation and suspension of license) gives some initial assurance that there may be a measure of fair functioning in the action of the Board. Unfortunately, the correlations in this area were only small to moderate.

**FIGURE 1:**
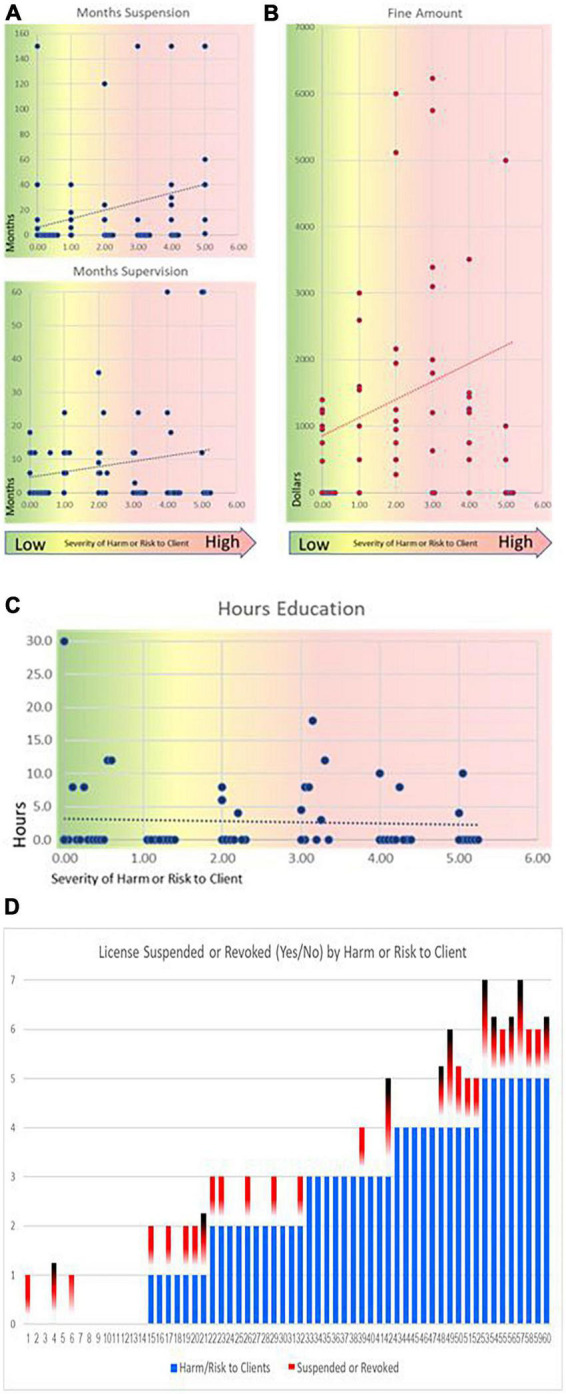
Correlation and outlier analysis. **(A)** Months of suspension and supervision. **(B)** Fine amount. The *x*-axis is harm or risk of harm and the *y*-axis is dollars (red dots) or months (blue dots). As the harm or risk of harm to the client increases the expectation is that the severity of the disciplinary actions taken by the board would also increase. Green banded points indicate no harm cases. Independent rating of total severity of offense did correlate with harm or risk of harm. However punitive actions by the board did not always correlate with harm or risk of harm (one outlier, $14,000, **(B)**, not shown). **(C)** The *x*-axis is harm or risk of harm and the *y*-axis is hours (blue dots). More severe punishments are at the top. Neither hours of continuing education nor total required supervisory hours significantly correlated with harm. Months of suspension weakly correlated with harm or risk of harm to clients. **(D)** Visualization of inconsistencies: although license suspension or revocation significantly correlated with harm or risk of harm, there remain cases where harm or risk of harm is very low, but licenses were suspended or revoked anyway. A red mark indicates a suspension and black/red is a revocation. Four revocations were permanent (longest black-red bars) and 6 could be appealed (shorter black-red bars). High harm or risk of harm are evident in all 8 cases with a score of 5 (farthest to the right), but also 2 conditionally revoked licenses had lower harm scores and 6 cases of suspensions with lower harm scores are also observed.

Furthermore, although the severity of punishment is consistent and prudent at the highest level of harm or risk of harm (i.e., Figure 1D), there is a near random application of punishment at levels below four. There are problematic inconsistencies that point to a second class of punitive actions of the Board, which indicates they may be acting contrary to a fair methodology in the cases of specific psychologists. Notably, we found no correlation between severity of offense or harm/risk to clients and a number of other punishments (fine amounts,^2^ hours of CE required, total hours of supervision required, or if final judgments listed the license as being remanded) which also tells us that the administration of these specific punishments is more arbitrary.

The Board is therefore in reality operating a two-tiered punishment system in which a more just ruling for egregious and obvious harm is given in regard to probation, suspension, and revocation, but this is overlaid onto a system in which additional punishments (fines, education hours, supervision hours, public humiliation) are doled out in an inconsistent way to psychologists with less severe infractions. According to the American Psychology Association, the principle of proportionality is a fundamental judicial principle maintaining that the severity of a punishment should be directly related to the seriousness of the crime. However, the data does not show proportionality in the case of these additional punishments.

Arbitrary punishments are also a symptom of bias and abuse of power. Such a two-tiered system would allow members of the Board to uphold a veneer of fairness while at the same time judge and punish psychologists based on overt negative feelings or implicit biases. This type of system can conceal injustice. The parts of the system which are administered in a moderately fair manner can be instrumentalized to provide justification for the unjust outcomes seen in other parts. For example, punishments can be doled out arbitrarily, such as the levying of fines by “feeling” or foregoing the public remanding of license for persons who may have a positive connection with sitting Board members. The KY psychology community is small, and personal or professional connections would be expected (e.g., “old boys” club). We found in this vein a bias against ECPs in the handing out of hours of continuing education. There are equal if not more reasons for older psychologists to be reeducated after a professional transgression, and yet as a class, ECPs appear to be punished more severely.

If a linear scale is utilized by the board to determine the severity of punishment, then for cases with higher risk or more serious infractions, it can be deemed as a “functional” and fair methodology. However, cases that deviate from this norm would require additional scrutiny. These outliers may reveal instances of unjust judgments. Specifically, when looking at particularly unjust cases these will be in the outlier cases in which the harm or risk of harm is low (green band Figures 1A–C and inconsistencies in Figure 1D above) but the punishment is severe, or conversely when the infraction is severe, and the punishment is not.

Inasmuch as the goal of punishment is to rehabilitate an errant psychologist in cases where the harm/risk (higher risk case or case with more serious infraction) was lower, these punishments failed in many cases. In our research, the disciplinary actions by the Board led many reprimanded psychologists who could have been rehabilitated to eventually leave the state or stop practicing altogether. We found that others with the worst infractions became the most difficult to locate (e.g., moved to another state and/or changed name), which thwarts any of the societal protection goals of publicly posting information about offenders.

In terms of license revocations, notably, several more senior psychologists accepted revocation and retired. Other psychologists had their licenses revoked by default for failing to respond to the Board. Based on what we know about the nature of trauma and the traumatizing effects of being accused (as outlined in the section “1.3. Ramifications of accusations of misconduct and disciplinary actions” and as elaborated upon below), we can expect that some accused psychologists would be unable to advocate for themselves due to overwhelming feelings of shock, grief, and shame (e.g., Kirkcaldy et al., 2022). Indeed, avoidance is a hallmark symptom of PTSD (Honig et al., 1999).

### 3.5. Discussion of interview findings

The interviews conducted revealed that the discipline process was highly distressing and even traumatizing in some cases. Kirkcaldy et al. (2022) noted “that psychological and physical symptoms will almost certainly occur in the wake of a complaint. If practitioners can be so severely affected by a complaint, it may have implications for their mental health and subsequent coping abilities if not adequately managed… practitioners in this position are at risk of becoming patients themselves, with the implication that a stressful complaint process could lead to problems with maintaining professional competency” (p. 406). Profound impacts on practitioner mental health were observed among all those interviewed, including those who withdrew their consent for their stories to be used. Reactions were trauma inducing and similar to those described in the section “1.3. Ramifications of accusations of misconduct and disciplinary actions.”

The qualitative data also reveals that some of the disproportionate disciplinary action recounted in the interviews may be due to biases and was perceived as such by the psychologists involved. As Kentucky has a history of racial bias in psychology, and as psychologists, we are aware that *everyone has biases*, the Board would need to have a system in place to mitigate biases to ensure that severities of disciplinary actions are proportional to the transgressions (American Psychological Association, 2010; Smith et al., 2015) and administered equitably and consistently. We think this is particularly salient for custody cases, as discussed below.

Another recurring issue that each interviewee expressed about the complaint and disciplinary process was the unresponsiveness from the Board. Throughout the complaint and disciplinary process, many interviewees stated that the Board does not respond to their requests and submissions in a timely or predictable manner. This unresponsiveness and unpredictability amplified anxiety and stress in an already stressful process. Another issue that made the complaint and disciplinary process unnecessarily traumatic was that letters or notices were sent during the holidays. As during the holidays, the Board would be closed and unlikely to respond to their emails or phones, so individuals who receive these letters cannot correspond to anyone about the letter itself or inquire about what next steps should be taken.

Moreover, during the investigation phase, many interviewees expressed that the investigator who conducted the interviews was often incompetent or unknowledgeable in the interviewees’ area of practice. Many of the investigator’s questions felt redundant or inappropriate. For example, one individual investigated by the Board expressed that in her area of practice, it was quite common to use exposure therapy, which might include taking their patients to the mall or to a restaurant as a part of their treatment process. However, because the investigator did not have knowledge of this empirically-validated treatment, they interpreted this well-supported therapeutic act as inappropriate (Deacon, 2012; Faber et al., 2022a,b). This process of misunderstandings and needing to educate evaluators on technical matters can cause unwarranted stress for a psychologist who must endure this line of questioning, and indeed this resulted in at least one report from an investigator that was inaccurate at the expense of the accused. For example, Francesca and Claire wrote a culturally-informed parental fitness report about their Black examinee that was ridiculed by the Board investigator as “unorthodox,” despite being reflective of current APA best practices (American Psychological Association, 2011).

### 3.6. Forensic, legal, and child custody cases: troubles unique to psychology

Complaints arise when psychologists venture into the area of forensic work “with unfortunate regularity” (Kirkcaldy et al., 2022, p. 9). Correspondingly, a number of cases (*N* = 15) that involved children were the impetus or focus of disciplinary actions by KBEP, and most involved the courts. These types of cases can be stressful for psychologists because of the high levels of distress and acrimony associated with the parents in this process. Due to the frequency of complaints, child custody work is often perceived as a high-risk area of practice (Bow et al., 2010). There is a dire shortage of clinicians willing to provide child evaluations to the court due to the (accurately) perceived professional risks of this important endeavor. As such, many courts are struggling to locate qualified evaluators, resulting in a backlog of cases.

These types of cases are one of the most frequent causes of reports against psychologists, to the point where the state of Colorado opted to stop considering complaints in such situations, and instead granted quasi-judicial immunity for child and family investigators, which can be a court-appointed mental health professional (Kirkland and Kirkland, 2001; Supreme Court of Colorado, 2011). Some states have protective laws, where psychologists who are involved in child custody proceedings are given immunity from civil suits, which have been very helpful for protecting those practitioners. For example, the Florida Psychological Association reported that prior to the implementation of a limited immunity law in Florida, *nearly 80 percent of all complaints* filed with the Florida Board of Psychology were for child custody evaluations (Koller, 2005).

In addition, studies have demonstrated that child custody cases are subject to gender bias which originates from stereotypes about gender roles; there is a general preference for maternal primary custody. As everyone is subject to implicit bias on both gender and racial axes, it is important to have mechanisms in place to assess and mitigate these psychological processes that produce biases which can blind impartial reasoning (Maldonado, 2017). In cases such as Claire’s, where there is a built-in disadvantage for the father both because he is male and because he is Black, higher scrutiny is warranted on the process by which he was denied an equitable evaluation because *prima facie* in such cases empirical evidence shows implicit biases tip the scales in favor of the White mother (Rachlinski et al., 2009; Costa et al., 2019).

Child custody and family court-related complaints are often quite complex and require expertise in the field. Informed by our qualitative research, and following recommendations from Kirkland and Kirkland (2001), we would suggest that cases related to child and family services not be heard by the Board without expert support being brought in, and/or unless the case involves clear and direct abuse of a patient/examinee by the psychologist (unrelated to the psychologists’ findings or purported professionalism). Various states have good faith statutes that protect psychologists from liability. These states include Florida, Georgia, and West Virginia. Kentucky, tellingly, has a statute granting only board members immunity for blanket good faith acts.

When it comes to cases that involve legal matters, we suggest the Board consider following the example of Florida, Ohio, and New Jersey to adopt the APA Specialty Guidelines for Forensic Activities into the statutes governing the work of the Board, to help provide clarity about the role of the Board vis a vis psychologist who provide expert opinions to the court (American Psychological Association, 2013). Although these laws are designed to protect custody evaluators who act in good faith from baseless legal actions and complaints filled with licensing boards, they do not provide protection to psychologists who are facing complaints filed against them with their state psychological association or the APA Ethics Committee.

## 4. Recommendations

### 4.1. Addressing power and privilege

As noted, there are concerns that punitive measures taken by the Board have been at times unduly harsh, inconsistently applied, and lacking in transparency. Our findings support these concerns and show that particularly for many early-career psychologists, the penalties that have been assigned indefinitely impact their professional career, their mental health and their well-being. Kentucky psychologists have the opportunity to consider the purpose and goals of the Board when it comes to early-career psychologists. Is it to guide, teach, and rehabilitate those who have erred, or is it to punish, humiliate, and shame? We believe it should be more of the former and less of the latter, and we hope that most psychologists would agree.

The findings of this investigation support the observations of Woody (2016), that “mental health practitioners serving in monitoring roles seem most prone to adopt a prosecutorial stance and accent the negative and eliminate the positive. The latter may reflect, at least in part, that mental health practitioners, unlike legalists, are not trained adequately in guarding against bias in professional issues.” (p. 199) Further, Woody notes that “new members of ethics committees and/or licensing boards acknowledge that their appointments led them to become highly judgmental and narcissistic about professional practices. For example, there is the risk of self-aggrandizement simply by virtue of being appointed by a membership vote, a political process, or a governor. This character flaw can spawn bias, prejudice, and discrimination against members of an out-group (i.e., a practitioner under scrutiny for wrongdoing); consequently, objectivity and justice may be subverted” (p. 199).

Furthermore, Boards such as KBEP and other psychology licensing boards often function as powers unto themselves, operating with little or no meaningful oversight. We posit that to better balance the need for oversight with the need to encourage learning and growth in the profession, the Kentucky Board of Psychology Examiners should: (a) define and outline different consequences for different career stages and for major and minor offenses; (b) ensure the investigation process for grievances is conducted by a neutral and well-qualified third party; and (c) provide a pathway for early-career psychologists who have been disciplined to prove their development and learning in order to clear their permanent record.

### 4.2. Defining different career stages and accountabilities

We recommend KBEP adopt a definition for early-career psychologists to provide predictability and consistency regarding relevant categorization and analysis in their duties going forward. For example, the Kentucky Psychological Association currently defines “early-career” as a psychologist who has been licensed for less than 7 years. It is important to have a clear definition for people falling into this stage of their career, since, as we have outlined, a professional’s stage of career and the type of alleged offense should have an impact on the kinds of remediations offered by the Board in the case of a complaint.

From our interviews, we found that early-career psychologists were more likely to continue self-education even after their graduation and entrance into the professional field. Early-career psychologists were more likely to incorporate cutting edge research into their practice and attempt to innovate the field by using the most recent evidence-based treatments. More remedial options are needed for early-career psychologists who may commit administrative errors due to confusion with the rules and legislation and lacking concrete experience with much of the administration of clinical practice. More protections are also needed against frivolous complaints so that ill-intentioned complainants are not able to take advantage of the relative inexperience and vulnerability of early-career psychologists, as the case of Claire for example.

If an incidence of misconduct is found to cause significant harm to a client, then the Board should discipline accordingly to ensure public safety is protected. However, if the offense is minor or administrative in nature and/or does not cause harm to a client or the public, then it would be reasonable to suggest that the Board take into consideration the early-career status of the psychologist in determining the consequences of their actions. In these cases, the Board should then avoid administering permanent-record disciplinary actions in favor of other restorative/rehabilitative remedies (see recommendations in Table 7).

### 4.3. Preventing undeserved disciplinary actions

See Table 6 for examples of such safeguards. As per the existing literature, we have found that poorly managed professional discipline processes can result in severe consequences for psychologists at all career stages, which may include humiliation, disgrace, loss of reputation, and loss of livelihood (Coy et al., 2016; Murphy, 2020; Kirkcaldy et al., 2022). Unfortunately, this sometimes happens on the basis of an unmerited complaint. Procedural due process protections apply to professional license actions to help prevent such errors, but the processes across states vary in the strength of the procedural safeguards they require in such hearings. When procedural safeguards are weak, it is far more likely that an undeserving professional will be unfairly and permanently harmed (Murphy, 2020). A preponderance of the evidence standard provides insufficient due process for licensed professionals in administrative disciplinary hearings when a state has no other safeguards in place. However, preponderance of the evidence may be appropriate when there are additional procedural safeguards in place—a standard termed “preponderance, plus” (Murphy, 2020).

**TABLE 6:** Psychologist protections for good faith.

States/Provinces	Laws/Jurisprudence/Act	Comments
Florida, USA	2022 Florida Statutes, Chapter 61, Part 1: General Provisions, s 61.122 https://www.flsenate.gov/Laws/Statutes/2022/0061.122	This statute outlines the presumption of good faith that is afforded to court appointed psychologists in child custody evaluations. While full immunity from prosecution is not provided, significant protections include a lack of anonymous complaint filing, and ensuring that a complaint be filed directly to the judge in the case. If a complaint is filed and it is proven that the psychologist did act in good faith, then the complainant must pay the psychologist’s legal fees
Georgia, USA	2022 Georgia Code, Title 19 - Domestic Relations, Chapter 9, Child Custody Proceedings, Article 1 - General Provisions ss 19-9-3 Establishment and Review of Child Custody and Visitation https://law.justia.com/codes/georgia/2022/title-19/chapter-9/article-1/section-19-9-3/	Under GA Code ss 19-9-3 (2022) (a)(7), the judge in a child custody case is authorized to order a psychological custody evaluation of the family or an independent medical evaluation. In such a circumstance, the appointed evaluator cannot be subject to civil liability resulting from any act or failure to act in the performance of their duties unless such act or failure to act was in bad faith. This statute provides full immunity protection to the psychologist, and there is no recourse if the evaluating psychologist can prove their assessment was done so in good faith.
West Virginia, USA	2022 West Virginia Code Article 7, Chapter 55, ss 55-7-21 Creating presumption of good faith for court-appointed licensed psychologists and psychiatrists conducting a child custody evaluation; method for assigning court and legal fees https://code.wvlegislature.gov/55-7-21/	Under WV Code ss 55-7-21 (2022) (a) a licensed psychologist or licensed psychiatrist who has been appointed by a court to conduct a child custody evaluation in a judicial proceeding shall be presumed to be acting in good faith if the evaluation has been conducted consistent with standards established by the American Psychological Association’s guidelines for child custody evaluations in divorce proceedings; (b) The complaint cannot be filed anonymously. Anonymous complaints are dismissed; (c) Any filings against court appointed psychologists/psychiatrists must be specific and have evidence otherwise they are dismissed; and (d) If psychologist/psychiatrist is entered into civil proceeding because of their court appointed child custody evaluation, then they will be reimbursed of all reasonable costs and attorney fees expended if it is proven they acted in good faith and in accordance with the APA. Similar to Florida, the psychologist can be sued but can recoup their legal fees if they can prove they acted in good faith

Currently, members of KBEP have an outsized position of power, and in their individual roles on the board can serve as complainant/instigator of case, witness, decision maker and disciplinarian. The lack of scrutiny of power-holding institutions such as these types of boards can create a space in which personal biases can play into the process, and injustices can occur without meaningful recourse for those affected. The overlapping roles can also result in a conflict of interest and become a barrier to due process. In the case of Claire, we noted that a Board member was appointed as a paid supervisor as part of a licensee’s discipline. As noted by Murphy (2020), licensing boards comprise colleagues who have ongoing trusting relationships. “The beliefs and recommendations of one member will inevitably influence others when there is no separation between functions” (Murphy, 2020, p. 959). A due process “plus” could take the form of adding additional seats to the Board (see below) to allow for the full removal of complainants/instigators from the rest of the hearing and still maintain quorum, and/or adding additional seats to the Board to allow for the removal of the committee who decides to bring the case forward from the adjudicatory hearing and still maintain quorum.

In the event that an error is made by the Board in terms of disciplinary actions, there must be a workable means of appealing the decision. Considering the shock and trauma of a negative finding, and the substantial cost of mounting a defense, the 30-day appeal window (noted in KRS Chapter 13B) seems inadequate. Indeed, it is unclear if anyone has ever been able to marshal the emotional and financial resources needed to appeal a Board decision (none were noted in any of the cases we reviewed). As such, the time should be lengthened to give an aggrieved party the necessary time to take this potentially corrective action.

One way we can determine if a practitioner is fairly or unfairly treated is by rating the severity of the offense and comparing it to the severity of the infraction. One would first determine the correlation between these variables in the entire population of disciplined practitioners, as was done in this study. The next task would be to plot the rating of the severity of the infraction by the rating of the severity of the discipline for the individual who was purportedly unfairly treated to see if they fell along the correlation line or if they are an outlier. An outlier would mean the person was punished unfairly, or differently than others.

### 4.4. Bringing in third parties to assess allegations of misconduct

An additional means of adding safeguards into the work of the Board is for the review and investigation process of grievances to be independently and expertly conducted. This would help bring greater accountability and expertise to the investigation and review process, as well as helping to manage the overall workload and accountability of the Board.

In the Kentucky Administrative Regulations, offenses are considered “grievances” (201 KAR 26:130, 2021). Grievances can be brought for consideration when there is “a clear and concise statement of the facts giving rise to the grievance” (201 KAR 26:130, 2021). When this has been submitted, the grievance is then reviewed by a complaint committee who, in the case of psychologists, is KBEP (201 KAR 26:130, 2021). We recommend an amendment to the process by introducing an independent complaint committee to review a grievance before it is escalated to the Board. This recommendation is in alignment with the practice in other states. In Massachusetts, for example, they have access to an independent committee that verifies the legitimacy of the complainant’s claim before continuing with the investigation process (251 CMR: 1, 2016). An independent committee can bring accountability, expertise, and efficiency to the process by offering a thorough review of the case by people with expertise in the particularities of the context, outlining a clear step-by-step investigation process, and collecting and providing preliminary evidence from both the complainant and from the psychologist(s) in question.

An independent complaint committee can help improve the investigation process by improving the accountability, transparency, and ethics of the review process. Also, the independent complaint committee could help avoid potential conflicts of interests because they are tasked with gathering information and have no considerable influence on the outcome of the case. Finally, the independent committee members would be composed of a diverse membership, and each member’s profile information should be readily and easily accessible online. The members of the independent committee should include psychologists with different interests and specializations, psychologists who are in private and research sectors, and psychologists of color and other equity-seeking groups. Individuals who are on the Board should not be a part of the independent committee because there will be a conflict of interest. Board members decide the verdict of the case and so they should only receive information about the case when all the facts have been gathered and confirmed by the independent committee for Board review and adjudication. With an independent committee as an interim step in the process, the Board can focus their efforts more on one of their core purposes: serving and building the practice of psychology in the state on behalf of the public, while an independent committee supports them in the work of assessing the nature of individual complaints. The final decision on a grievance would still lie with the Board, but the independent committee can help filter out relevant and irrelevant claims which improves efficiency, transparency, and impartiality for all involved.

### 4.5. Ensuring rehabilitation: early-career missteps should not be permanent or career-ending

The Kentucky Board of Examiners of Psychology should provide opportunities for early-career psychologists to remove disciplinary actions from their private record if they receive an admonishment. A psychologist that makes a mistake early on in their career should not be held to the same weight as a psychologist who has more experience; an early-career psychologist is most likely going to make missteps as it will be their formative years and they will have less experience compared to a more senior psychologist. Indeed, if every early career practitioner in Kentucky who made an error was prevented from practicing, there would be no psychologists in the state. As such, for early-career individuals who have received disciplinary actions or private admonishments, we recommend a process to remove them from their record once appropriate remediations have been fulfilled. This process can be completed through a course or required program or through good behavior after a designated period. Alternatively, private admonishments could have an expiration date, being removed automatically after a given period of time.

Another potential middle ground that the Board could explore is revising the way that the disciplinary action of private admonishment is administered. Currently, the Board can impose a private admonishment or a more severe disciplinary action against the psychologist (see Table 1). The Kentucky Revised Statutes explains that the private admonishment is not to be disclosed to the public and it does not constitute as a disciplinary action (Kentucky Board of Examiners of Psychology, 2022). However, in practice, the private admonishment is a permanent mark on a licensed psychologist’s record in Kentucky that inadvertently acts as a form of punishment. It is a consistent reminder that the Board has been surveying their actions long after the investigation and decision has concluded. Disclosing the private admonishment to prospective out-of-state employers might cause unease and uncertainty in their assessment of a candidate’s application, while withholding the private admonishment can cause discomfort in the psychologist because they may feel they are not being completely honest with their employer. It can cause an early-career psychologists’ immense stress and anxiety knowing that they have a stain on their record that cannot be removed.

A further line of inquiry could be to explore parameters and boundaries around major and minor offenses. Currently, the Kentucky Revised Statutes do not define major or minor offenses. It provides a list of conduct that they deem to be offensive and can lead to disciplinary actions (KY §319.082, 2019). These offenses range from minor, “unlawfully failed to cooperate with the Board by not appearing before the Board at the time and place designated,” to ambiguous, “violated any state statute or administrative regulation governing the practice of psychology…” (KY §319.082, 2019). We recommend KBEP define minor and major offenses and the potential consequences that each hold. By providing succinct definitions between minor and major offenses, the Board will be able to clarify the expectations and consequences for breaking an offense, creating a stronger relationship between the Board and the psychologists that they oversee. For example, the Wisconsin Psychology Examining Board defines a “minor violation” as “…no significant harm was caused by misconduct of the credential holder,” such as failing to keep good notes (Legislative Reference Bureau, 2022). The lack of clarity between minor and major offenses in Kentucky can cause anxiety and fear in psychologists who receive threatening notices from the Board; providing a definition between the offenses can help ease their minds in this trauma-inducing process.

A more appropriate consequence for early-career psychologists who commit minor violations would be to require them to complete continuing or additional education courses or provide a letter of warning. In Wisconsin, if the psychologist is found to have committed a minor offense, where the consequences of the actions are unlikely to cause significant harm and the continued practice would present no immediate danger to the public, then the likely result of prosecution would be a reprimand or a limitation requiring the credential holder to obtain additional education (Legislative Reference Bureau, 2020). In California, if the psychologist is found to have committed a minor offense, the Board might mediate an agreement between the complainant and the psychologist, issue the psychologist a letter of warning, or set up an educational conference between the psychologist and an expert case reviewer and/or Board member (Department of Consumer Affairs, 2022).

### 4.6. Providing support and advocacy

Being formally accused of unprofessional conduct by a licensing board is a life-changing and potentially career-ending event. Because accusations of wrongdoing create a stigma that therapists are unprepared to handle, obtaining support is vital (Coy et al., 2016). Due to the potentially traumatizing nature of the investigation and disciplinary process (e.g., Kirkcaldy et al., 2022), we recommend that KPA invest in the creation of a diverse group of knowledgeable peer advocates to provide ongoing support and administrative guidance for any psychologist accused of wrongdoing. The role of these advocates is not to replace the role of an attorney, but rather to assist and support the accused through the process. Our interviews showed that a significant amount of the stress and trauma generated by the actions of the Kentucky Board revolved around the difficulties in reaching the Board to get information about their situation, and the lack of communication, orientation or conversation around the charges being laid against them. Further, peer support could reduce the shame and trauma of being accused, and help psychologists maintain their professionalism and perspective during a trying process.

Given the potential damage that these processes can inflict on a person’s professional life and psychological well-being, we also suggest that there be a clearer process developed for members of the KPA and those governed by KBEP to be able to bring a complaint toward an individual Board member. Right now, removing a Board member involves the Governor reviewing a recommendation for removal from fellow Board members (KRS 319.020), based on “incompetence, neglect of duty, or malfeasance in office.” As the Board’s accountability to the community is strengthened in regard to the performance of their duties, so too should the publicly available mechanisms to hold them to account, individually and collectively. While Board members are immune from personal liability in the good faith execution of their duties (KRS 319.118), those governed by the Board should have a clear mechanism for bringing a complaint to the Governor or another entity if they experience harm due to a Board members potential incompetence, neglect of duty, or malfeasance.

### 4.7. Strengthening the board’s equity and diversity engagement

Based on the data gathered, it is apparent that KBEP often takes an authoritarian, fear-based approach to its duties that is oppressive toward psychologists in the state. This includes a lack of accompaniment and support in navigating the Board’s review process, treating people accused of administrative violations as equal to serious misconduct accusations, frequently withholding information and communication with people under review, and not providing opportunities for learning, reparations, and remediation. All of this creates a blanket culture of accusation, confusion, fear, and punishment that undermines the purpose and function of the Board, with a negative impact on the psychology community in Kentucky.

Further, there were many troubling issues surrounding diverse and stigmatized identities that emerged in the qualitative data. Notably, there were numerous concerns voiced about discrimination and prejudice in the processes. Considering the data and the APA guidelines, in cases where race or other marginalized identities are a factor, the Board should be able to show its processes in regard to how it worked to mitigate its own implicit bias as well as how it explicitly considers racial issues in such cases, as like it or not, they will have an influence on the outcome (Maldonado, 2017).

Two of the authors of this paper (EW and AB) were scheduled to present this report to the KPA annual convention in Louisville, KY in 2022 with a colleague joining as a discussant. However, a preliminary report of the findings in this article was leaked in advance of the conference, which made its way to members of KBEP who were displeased. Rather than attend the scheduled conference presentation or contact the authors to voice their concerns, they forced the KPA to have the presentation canceled with threats of legal action. Although this demand censored diverse voices and represented an infringement of academic freedom, the KPA was too intimidated to resist this injustice. These actions serve as a model example of the type of power abuse reported by those we interviewed and others who were supportive of this investigation but too frightened to come forward publicly. The retaliation of the Kentucky Board against its own state psychology association, KPA, must be seen as a dramatic turn and astonishing response to the public airing of material contained within this report.

For these considerations, experts with experience in equity, implicit biases, and cultural competency should be brought in as consultants to train the KBEP while also providing regular oversight. This would help to ensure that cases in which race, sexual and gender minority status, or culture are a factor are not unduly influenced by biases.

### 4.8. Equitable composition of the board

In the course of our investigation, a recurring theme was the problematic nature of the composition of KBEP. Although the current makeup of the Board maintains a balance of gender, all nine members (100%) are White-presenting, which is not representative of the racial makeup of the state of Kentucky as of 2020 (White alone 61.6%; Black alone 12.4%), nor of those who practice psychology in the state (United States Census Bureau, 2021). Implementing a specific and general provision into the current legislation to ensure more diversity in the composition of KBEP would help ensure the protection of marginalized psychologists accused of misconduct, better serve the public and the community of psychologists it oversees and improve equity within the functioning of the Board.

The profession of psychology was originally developed with a racist framing. Psychologists have subjected persons of color (e.g., those of African descent and Indigenous people) to abusive treatment, experimentation, victimization in the name of science, along with racialized theories that attempted to justify their subordinate status (Guthrie, 2003; Dupree and Kraus, 2022; Williams et al., 2022). To illustrate racialized difficulties of practicing psychology in Kentucky is the story of Robert Val Guthrie, who was the only African American in the psychology master’s program at the University of Kentucky in 1960 (Smith et al., 2015). He stated that his primary objective was to get his degree and “get the hell off campus” because of the racism he was experiencing (Smith et al., 2015). He later penned the seminal book entitled *Even the Rat was White: A Historical View of Psychology*. To this day, the number of Black practicing psychologists in Kentucky is abysmally low. At a recent meeting, the APA Council of Representatives (2021) adopted an apology for the organization’s role – and the role of the discipline of psychology – in contributing to systemic racism. The APA acknowledged that it “failed in its role leading the discipline of psychology, was complicit in contributing to systemic inequities, and hurt many through racism, racial discrimination, and denigration of people of color, thereby falling short on its mission to benefit society and improve lives” (American Psychological Association, 2021). Yet into current times, many of these problems remain.

As previously noted, everyone has biases, which can be implicit or explicit (e.g., Gran-Ruaz et al., 2022). The literature indicates that clinicians deemed “foreign” may receive harsher penalties than those considered part of the in-group (Humphrey et al., 2011). Therefore, it is necessary to put systems in place to mitigate such biases whenever possible. As the field of psychology becomes more diverse, we are seeing increasing diversity among early-career psychologists (Pedrotti and Burnes, 2016). Given that the group dynamics of the licensing Board can be likened to a jury when conducting disciplinary hearings, the same types of in-group biases can be expected to emerge that would disadvantage psychologists of color. As such, deliberate anti-racist approaches should be utilized (Williams et al., 2023).

Our participants and our own analysis found that the Board did not adequately represent the population of Kentucky nor the clients they treat, in part due to the racial makeup of the Board. There were concerns by several interviewees that racial biases were a factor in their experiences. One critical way to address this perception is to promote diversity within the Board by ensuring that the Board has seats dedicated to different equity-seeking groups, such as racialized people, religious minorities, and members of the LGBTQ + community. This approach of having dedicated seats for racialized and equity-seeking groups can help cultivate more trust in the process, a more representative board, and also keep the importance of diversity and community representation at the forefront of how the Board operates. It is important to note as well that having dedicated seats does not mean that these are the only seats for equity-seeking groups. People of color could occupy all of the Board positions, but this approach ensures that a bare minimum level of representation is established. This practice of dedicated seats has been implemented by other Kentucky boards, and notably by the Board of Trustees of both the University of Kentucky and University of Louisville. They require that the members appointed by the Governor must reflect a proportional representation of the minority racial composition of the Commonwealth and cannot be less (Ky. Rev. Stat. §164.131, 2017; Ky. Rev. Stat. §164.821, 2017).

Diversity can also be promoted by using more holistic and progressive recruitment and selection practices. As the KPA administers the nomination of Board members, it could put focused effort into advocating for a more diverse slate of nominees. For example, the KPA can be more deliberate about where it advertises its call for nominations, and what kinds of information it shares in its postings to help encourage people from diverse backgrounds to apply (such as saying that new members will be mentored by an existing member, governance training will be provided, the Board needs and welcomes new and diverse perspectives on the practice of psychology in Kentucky, special stipends for members of marginalized groups, etc.).

Finally, we believe that there needs to be diversity within the Board members’ professional areas of expertise. Currently, the Kentucky Board is mainly composed of psychologists working in the legal and insurance arenas, and as such the composition of the Board does not reflect the diversity of professionals that are accountable to the Board. We also believe that an early-career psychologist should be on the Board. KBEP recognizes the need for the opinion of the public by appointing a citizen at large and should extend the need for different perspectives within the practice of psychology in the state by appointing a new early-career position, as well as a university psychology researcher. This will ensure that there is a specific person on the Board who can provide important insight and perspective to Board decisions relating to this specific phase of every psychologist’s career, as well as ensuring the Board is on top of the latest science, research, and up-to-date practices (e.g., culturally-informed assessments, evidence-based treatment approaches).

### 4.9. Diversity training

Another way of improving the work of the Board is to raise awareness of unconscious bias, especially given the critical power differentials in decision making (Pedrotti and Burnes, 2016). One way to do this is by requiring Board members to engage in anti-bias training. For example, in Michigan, almost all professionals, including psychology professionals, under the Michigan Public Health Code are required to take Implicit Bias Training as a condition for their initial licensure or registration, as well as every time they renew their license/registration (Mich. Admin. Code R. 338.7001-7004, 2021). There are several interventions that have been shown to reduce racial bias, which should be combined with ongoing learning to keep knowledge current (Williams et al., 2020, 2022).

### 4.10. Limiting power: term limits and complaints

A final suggested amendment to current legislation is to modify the time limits that individuals can serve on the Board. Currently, members are permitted to stay on the Board for 4 years and cannot serve more than two consecutive full terms. We believe that this is not in accordance with best practice, as neighboring states such as Georgia limit Board members to a singular term of 5 years. Furthermore, through our qualitative research, our participants have noted that more turnover in Board members would help bring new perspectives and updated thinking into the work. The Canadian Psychological Association (2018) found that limiting the years that one can serve on a Board promotes fresh and diverse perspectives. With this in mind, KBEP should consider limiting people’s engagement to one term. And as discussed above, these new shorter-term limits could be accompanied by an accountability mechanism for the public to be able to bring a complaint to the Governor for an individual Board member’s incompetence, dereliction of duties, or malfeasance.

### 4.11. Humanistic approach

In terms of how the Board works, as mentioned above, a significant portion of the problematic and damaging psychological effects of the Board’s engagement with accused psychologists could be tempered by providing a more human-centered and compassionate approach to communication and accompaniment in the disciplinary process. Information is power, and the Board has not shared that power equitably with the people it is meant to accompany and serve. The effects are often felt particularly strongly by the most vulnerable in the psychology community, including psychologists from marginalized groups and early-career psychologists. Treating people with kindness and respect, providing clear information about the process that is unfolding, being available to answer questions as they arise, and sharing resources to help people understand and orient themselves within an already very stressful and potentially career-threatening situation are all basic ways to help improve the way the Board operates with its members and lessen the damage being caused to the profession.

All the recommendations (Table 7) are offered in support of deepening KBEP’s legislative oversight role in the state, as well as in support of their overarching mandate to help foster a vibrant, safe, effective and accountable field of psychological practice in Kentucky. While accountability and public safety are of utmost importance in the practice of psychology, so too is the need to increase the sheer number of trained psychologists to meet the increasing demand for mental health services.

**TABLE 7:** Recommendations for improved policies and procedures.

Recommendation table	
Legislative changes	Limit Board members’ service to one term
	Require that Board members appointed by the Governor reflect a proportional representation of the racial composition of the Commonwealth.
	Create a permanent Board seat for an “Early Career Psychologist” and define this term in the legislation.
	Create a range of clear processes for different kinds of complaints that take into account a range of external factors as well as harm/risk assessment to the client and society (i.e., administrative issues should be treated differently than complaints connected to court hearings, or complaints that relate to patient exploitation or abuse).
	Create a mechanism for psychologists with disciplinary actions on their record to have these removed.
	Create a mechanism for complaints to be made toward individual Board members for their discipline or removal, that does not involve the sitting Board as part of the decision process.
	Extend the amount of time disciplined psychologists have to appeal the Board’s decisions from 30 days to 1 year.
Policy/Procedure changes for KBEP	All complaints should be sent initially for external evaluation to determine the merits of the complaint before being added to the Board’s agenda for final determination.
	If a complaint arises from the Board itself, it should include a full written account of the issue, origination of the concern, and name of the Board member advancing the complaint (just as would a complaint arising from a member of the public).
	Separation of those Board members who decide to bring a case to prosecution and those making decisions at the adjudicatory hearing.
	Use investigators with expertise in the professional area of the accused.
	Except for direct client exploitation, early-career psychologists receive no more than a private admonishment or continuing education for a first offense, with no disciplinary (reportable) findings.
	Provide and require anti-bias and anti-racism training for all Board members.
	Revise wording of letters and communications from the Board to accused psychologists to be less threatening, keeping in mind they are innocent until proven guilty.
	Send disciplinary notices, information, and communications around Board processes with reasonable timelines and at times of the year when people will be available to respond in a timely way.
	Ensure that someone can respond to all inquiries to the Board within 24-h.
Policy/Procedure changes for KPA	Establish a peer advocate community to provide additional accompaniment and support to any accused psychologists going through a disciplinary process.
	Work to elicit a more diverse slate of nominees for appointment to the board. Diversity should consider race, ethnicity, religion, training, and occupation. Given the extra service work often required by people with marginalized identities, KPA could provide additional support to these members if elected.
	Work with the Board to develop a system that fairly and consistently penalizes wrongdoing in a manner proportional to the offense.

Notably, all persons interviewed in this study who were currently residing in Kentucky expressed a deep fear of reprisal from the Board, and none felt able to risk coming forward publicly with their stories. Further, although many Kentucky psychologists assisted with this project, none were willing to be publicly acknowledged out of fear of being targeted by the Board and jeopardizing their livelihood. Given that accusations against psychologists may arise from the Board without explanation (as we saw in Daria’s case), and outcomes are uncertain, these fears are not unjustified. This culture of fear makes it impossible for psychologists in the state to feel safe to collaborate with the Board or advocate for positive change for the profession and people of Kentucky. There is a need for a more humane and equity-based approach that goes beyond procedural or policy changes.

## 5. Study limitations

Despite best efforts, there are some limitations to this investigation. Low correlations between ratings of danger to the public and disciplinary actions could be due in part to the messiness of the data. It would help to have more standardization in terms of the data collection and categorization to make such analyses more straightforward for future evaluations (Liu and Bell, 2017). The recommendations offered have all been implemented to different degrees in various licensing boards and/or organizations, but more focused research would be needed to determine precisely how effective they are in advancing equity in psychology licensing boards, especially when implemented in aggregate.

## 6. Conclusion

Although new legislation is likely the best response to these concerns to ensure lasting change, we recognize that such changes can take time, whereas new policies can be more quickly implemented. The issues faced by KBEP, and the Kentucky psychology community did not happen overnight, but much can be done to start turning the tides in a more positive and constructive direction. Just as the APA apologized for its role in promoting racism in psychology (APA Council of Representatives, 2021), we humbly urge the KPA to take responsibility for its role in creating a homogenous and punitive Board and perpetuating or tolerating the harmful, unjust, and biased practices described herein. In the acknowledgment of harm and the plans to do better reside the seeds and momentum to create positive changes and an improvement in services for everyone in the state of Kentucky.

We believe that if the Board promoted fairness and diversity as part of how it operated and conducted itself administratively, the psychology community in Kentucky will be better served by this inclusive culture and approach, and psychologists in the state will be better equipped and supported to treat the diversity of people they serve. The field of psychology is constantly evolving and benefits greatly from the inclusion of new and underrepresented voices who can contribute to the development of the practice of psychology, and the equitable and effective provision of these essential social services in our communities. The methodologies utilized in this report may provide a framework to better understand the disciplinary work of other profession-based oversight Boards in KY or other states. Until such a time as KBEP can implement more equitable and conscientious functioning, individuals finding themselves persecuted by said Board may have no recourse for relief. We hope that larger bodies, such as the APA, the Association of State and Provincial Psychology Boards (ASPPB), and the Kentucky Governor’s office, will support those who might be negatively impacted by the conditions outlined in this report.

The KBEP has operated with a lack of meaningful oversight as complainant, jury, and judge for psychologists in the state of Kentucky. Our findings are consistent with the operation of a two-tiered system within their oversight activities: one in line with harm or risk of harm to clients, and another which has levied punishments in a way inconsistent with the principles of proportionality. The Board must therefore examine its policies, composition, practices and values to ensure they are fully meeting their legislated mandate, while also ensuring that everyone in Kentucky has the opportunity to access high-quality mental health services. There are changes needed in order for the Board to take full advantage of the benefits that a more inclusive and supportive culture would provide to professional psychology in the state, in order to cultivate a flourishing psychology practice in Kentucky and to better serve those citizens who can benefit from the mental health services psychologists provide.

## Data availability statement

The raw data supporting the conclusions of this article will be made available by the authors, without undue reservation.

## Ethics statement

The studies involving humans were approved by the REB Research Ethics Board of Ottawa University. The studies were conducted in accordance with the local legislation and institutional requirements. The participants provided their written informed consent to participate in this study. Written informed consent was obtained from the individual(s) for the publication of any potentially identifiable images or data included in this article.

## Author contributions

SF and AB contributed primarily to the text. EW contributed to the legal analysis, text referring to legal issues, and references. SF created the figures. All authors contributed to the analysis and the article and approved the submitted version.

## Footnotes

1
www.psy.ky.gov

2 *Fines are not supposed to be punitive, although based on the readings of the cases, and statistical analysis, it appears they are often used that way.
